# REV1 Inhibition Enhances Radioresistance and Autophagy

**DOI:** 10.3390/cancers13215290

**Published:** 2021-10-21

**Authors:** Kanayo E. Ikeh, Erica N. Lamkin, Andrew Crompton, Jamie Deutsch, Kira J. Fisher, Mark Gray, David J. Argyle, Won Y. Lim, Dmitry M. Korzhnev, M. Kyle Hadden, Jiyong Hong, Pei Zhou, Nimrat Chatterjee

**Affiliations:** 1 Department of Microbiology and Molecular Genetics, University of Vermont, Burlington, VT 05405, USA; Kanayo.Ikeh@uvm.edu (K.E.I.); Erica.Lamkin@uvm.edu (E.N.L.); Andrew.Crompton@uvm.edu (A.C.); Jamie.Deutsch@uvm.edu (J.D.); Kira.Fisher@uvm.edu (K.J.F.); 2 The Royal (Dick) School of Veterinary Studies and Roslin Institute, University of Edinburgh, Easter Bush, Roslin, Midlothian, Edinburgh EH25 9RG, UK; Mark.gray@ed.ac.uk (M.G.); david.argyle@ed.ac.uk (D.J.A.); 3 Department of Chemistry, Duke University, Durham, NC 27708, USA; won.young.lim@duke.edu (W.Y.L.); jiyong.hong@duke.edu (J.H.); 4 Department of Molecular Biology and Biophysics, University of Connecticut Health Center, Farmington, CT 06030, USA; korzhniev@uchc.edu; 5 Department of Pharmaceutical Sciences, University of Connecticut, 69 N Eagleville Rd, Unit 3092, Storrs, CT 06269-3092, USA; kyle.hadden@uconn.edu; 6 Department of Biochemistry, Duke University School of Medicine, Durham, NC 27710, USA; peizhou@biochem.duke.edu; 7 University of Vermont Cancer Center, University of Vermont, Burlington, VT 05405, USA

**Keywords:** translesion synthesis, radioresistance, autophagy, REV1, ionizing radiations, etoposide

## Abstract

**Simple Summary:**

Cancer resistance to therapy continues to be the biggest challenge in treating patients. Targeting the mutagenic translesion synthesis (TLS) polymerase REV1 was previously shown to sensitize cancer cells to chemotherapy. In this study, we tested the ability of REV1 inhibitors to radiation therapy and observed a lack of radiosensitization. In addition, we observed REV1 inhibition to trigger an autophagy stress response. Because reduction of REV1 triggered autophagy and failed to radiosensitize cells, we hypothesize REV1 expression dynamics might link cancer cell response to radiation treatment through the potential induction of autophagy.

**Abstract:**

Cancer therapy resistance is a persistent clinical challenge. Recently, inhibition of the mutagenic translesion synthesis (TLS) protein REV1 was shown to enhance tumor cell response to chemotherapy by triggering senescence hallmarks. These observations suggest REV1’s important role in determining cancer cell response to chemotherapy. Whether REV1 inhibition would similarly sensitize cancer cells to radiation treatment is unknown. This study reports a lack of radiosensitization in response to REV1 inhibition by small molecule inhibitors in ionizing radiation-exposed cancer cells. Instead, REV1 inhibition unexpectedly triggers autophagy, which is a known biomarker of radioresistance. We report a possible role of the REV1 TLS protein in determining cancer treatment outcomes depending upon the type of DNA damage inflicted. Furthermore, we discover that REV1 inhibition directly triggers autophagy, an uncharacterized REV1 phenotype, with a significant bearing on cancer treatment regimens.

## 1. Introduction

Intrinsic and acquired resistance to DNA-damaging cancer therapy is a persistent clinical challenge that ultimately limits successful clinical outcomes in patients [1]. Recent evidence suggests that a possible strategy to sensitize tumors and reduce chemotherapy resistance is to inhibit the mutagenic translesion synthesis (TLS) pathway by targeting REV1 TLS polymerase [2,3,4,5]. Translesion synthesis is a DNA damage bypass process involving a set of specialized DNA polymerases that collectively tolerate DNA damage and cause mutations [1]. REV1 plays a central role in this process by engaging in protein–protein interactions via two distinct interfaces at its C-terminus domain (CTD) [6,7]. Small molecule inhibitors targeting these interfaces can effectively inhibit mutagenic translesion synthesis and suppress tumor growth [3,4,5]. Remarkably, during a chemotherapy regimen, REV1 inhibition also switches the biology of cisplatin-dependent cell death response from apoptosis to senescence and triggers an immune response in treated cells [8]. It is unknown how exactly REV1-inhibition triggers senescence.

Radiotherapy is one of the mainstay treatment modules for roughly half of all cancer patients, typically involving high energy X-rays at doses of between 1 and 2 Gy per treatment fraction [9]. Despite promising initial success with radiotherapy in inhibiting tumor growth, relapse of the incumbent tumor and the subsequent requirement for a higher dose of radiation results in patient fatality. Several studies in the past few years have explored adjuvant therapies that might facilitate the continued sensitivity of the tumor to radiotherapy, but the underlying complex heterogeneity of the tumor itself has not allowed appreciable success. Mechanisms conducive to therapeutic resistance to radiation range from enhanced DNA double-strand break repair (DSBR) to altered expression of DNA damage signaling, in addition to the hypoxia-dependent protective effects from surrounding tissue [10]. Furthermore, radioresistance biomarkers have continued to be discovered within the clusters of transcriptional regulation of DNA metabolic processes, inhibition of apoptosis, response to hypoxia, and DNA repair. Because REV1 functionally links DSBR with enhanced mutations and REV1 inhibitors suppress apoptosis and trigger senescence to sensitize cancer cells to chemotherapy [8,11,12], it is unknown whether REV1 may similarly sensitize cancer cells to radiation therapy and prevent radioresistance.

This study tested whether REV1 inhibition via CTD-specific small molecule inhibitors sensitizes cancer cells to radiation treatment. In contrast to their chemosensitization effects [2,3,4,5,8], REV1 inhibition failed to sensitize cancer cells to ionizing radiation. We confirmed the lack of radiosensitization by using five different REV1 inhibitors, varying the doses of REV1 inhibitors, testing physiologically relevant increasing ionizing radiation doses, and examining radioresistant cell lines. Unexpectedly, we discovered that REV1 inhibition by small molecule inhibitors triggered autophagy, which is known to cause therapy resistance in cancer cells under certain conditions. Further, we found a narrow range within which an autophagy inhibitor might aid in sensitizing IR and REV1 inhibitor-exposed cancer cells [13,14]. These results highlight an unexpected new function of REV1 which is beyond translesion synthesis in determining therapy resistance, with critical cancer therapy outcomes.

## 2. Results

### 2.1. REV1 Inhibition Does Not Sensitize Cancer Cells to IR

**Figure 1:**
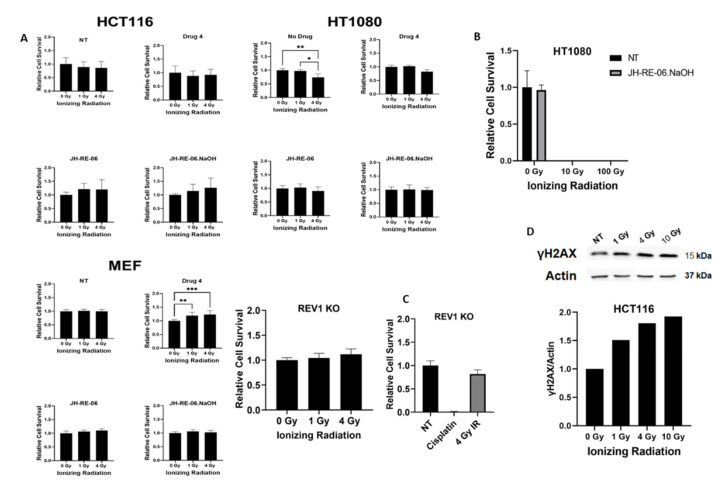
REV1 inhibition does not sensitize cancer cells to ionizing radiation. (**A**) Relative cell survival of HCT116 (colorectal), HT1080 (fibrosarcoma), mouse embryonic fibroblasts (MEF), and REV1 KO (knockout; MEFs) cells in response to increasing amounts of ionizing radiation at doses of 1 and 4 Gy and REV1 inhibitor drugs at a 1 μM concentration: 4 (7922759), JH-1 (JH-RE-06), and JH-2 (JH-RE06.NaOH). (**B**) Relative cell survival of HT1080 cells with 0, 10, and 100 Gy. (**C**) Relative cell survival in REV1 KO cells after treatment with increasing doses of IR at 0, 1, and 4 Gy (left graph) and with 10 mM cisplatin and 4 Gy of radiation (right graph). (**D**) Western blot showing γH2AX in HCT116 cells treated with 1, 4, and 10 Gy of radiation at 24 h. Graph shows the relative quantification of the Western blots. *p*-Values are * *p* < 0.05, ** *p* < 0.01, and *** *p* < 0.001. Error bars represent standard deviations. *p*-Values were calculated by 2-way ANOVA. *N* = 6 for all values.

REV1 protein plays a crucial role in the DNA damage bypass process by functioning as a scaffolding molecule that facilitates protein–protein interactions with other TLS polymerases via its CTD [1]. Previously, REV1 inhibitors that targeted specific interfaces of this CTD were shown to sensitize cancer cells to chemotherapy treatment, which suggested that the REV1-dependent DNA damage bypass of chemotherapy-induced damage was the cause of chemoresistance [2,3,4,5,8]. Whether REV1 inhibition would similarly sensitize cancer cells to radiation treatment is currently unknown. Because REV1 plays a role in the DSBR pathway and suppresses apoptosis—processes known to be involved in radioresistance—we hypothesized that REV1 inhibition might sensitize cancer cells to radiation treatment [14]. To test this hypothesis, we exposed mouse embryonic fibroblasts (MEF), HT1080 (fibrosarcoma), HCT116 (colorectal), and REV1 KO (knockout MEF) cells to physiologically relevant increasing doses of IR (1 and 4 Gy) and tested five different REV1 inhibitors that target different interfaces at a 1 μM dose. These drugs were: 4 (7922759), 5 (7587885), and 6 (7127492) which target the RIR (REV1-interacting region) interface; and drugs JH-1 (JH-RE-06) and JH-2 (JH-RE06.NaOH), which target the REV7 interface of REV1 (Figure S1) [3,4]. The RIR-specific drugs successfully inhibit REV1’s ability to interact with TLS polymerases—POL η, POL ι, and POL κ. JH-RE-06 specifically induces dimerization of the REV1 CTD that precludes REV7 from its binding pocket. The REV1/REV7 interface is also considered to be more mutagenic than the RIR interface as the RIR polymerases function redundantly. All these drugs were previously reported to suppress chemoresistance in cancer cells, where the sensitization effects of the REV7 inhibitor were stronger than those of the RIR inhibitors [3,4]. Additionally, the drug JH-RE-06.NaOH is a newer and more stable version of JH-RE-06, with an additional NaOH moiety. Using colony survival assays, our results showed no increased sensitization of these IR-exposed cancer cells to REV1 inhibitors (Figure 1A, Figures S2 and S3). Colony survival assays measure the relative potential of exposed cells to proliferate and form colonies. Similarly, we tested the ability of REV1 inhibitors to sensitize the cancer cells to increasing doses of IR in the cytotoxicity assays and observed no synergy in cytotoxicity (Figure S3). Additionally, the MEF REV1 KO cells were not sensitized to 1 or 4 Gy in colony survival and cytotoxicity assays, unlike the cisplatin (chemotherapy) sensitization observed in REV1 KO cells (Figure 1C and Figure S3). In fact, we consistently observed an increased proliferation potential post-treatment with REV1 inhibitors in all our assays, suggesting REV1 inhibition played a cytoprotective role in IR-exposed cells. The relative lack of toxicity observed here is in line with the previously published trends in these specific cell lines [15,16,17,18]. We also tested higher doses of IR—10 and 100 Gy—on HT1080 cells but were unable to note synergy due to higher toxicity of the radiation (Figure 1B). We tested whether there was any discernible indication of double-strand breaks from IR, which is the physiological response in cells, and observed induction of γH2AX in HCT116 cells, as shown in Figure 1D.

**Figure 2:**
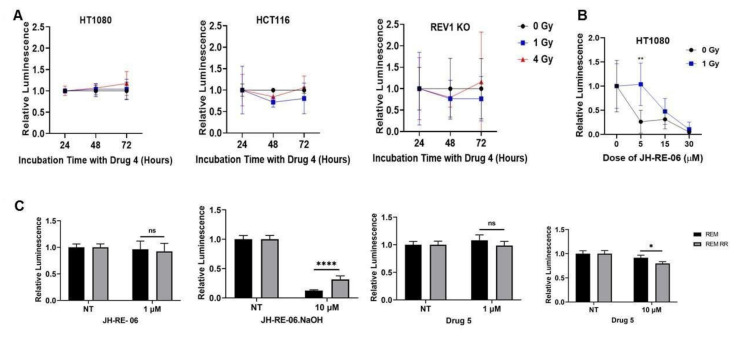
REV1 inhibition does not sensitize cancer cells to ionizing radiation. (**A**) Graphs show relative luminescence with increasing incubation times (24, 48, and 72 h) with drug 4 (7922759) and increasing doses of IR (0, 1, and 4 Gy) in the HT1080 and HCT116 cell lines. Also shown are relative luminescence intensities in MEF REV1 KO cells after exposure to 1 and 4 Gy ionizing radiation and incubation for 24, 48, and 72 h. (**B**) Relative luminescence in HT1080 treated with 0, 5, 15, and 30 μM of JH-RE-06 with 1 Gy of IR exposure. (**C**) Relative luminescence in REM and REM RR cells in response to treatment with JH-RE-06, JH-RE-06.NaOH, and drug 5 (4053831). *p*-Values are * *p* < 0.05, ** *p* < 0.01, and **** *p* < 0.0001. Error bars represent standard deviations. *p*-Values were calculated by 2-way ANOVA. *N* = 6 for all values.

To rule out the possibility that a longer time frame of REV1 inhibition was necessary to sensitize cells to IR, we exposed HT1080, HCT116, and MEF REV1 KO cells to increasing doses of 1 and 4 Gy of IR and 1 μM REV1 inhibitors 4 (7922759) and JH-RE-06 and measured relative differences in cytotoxicity at 24, 48, and 72 h. We found no significant increase in synergy in cytotoxicity with regard to increasing exposure times to REV1 inhibitors 4 and JH-RE-06 (Figure 2A and Figure S4). The relative luminescence across different exposure times in treated cells was unchanged from the non-treated controls. These results suggested that increased exposure time to REV1 inhibitors did not sensitize cells to radiation treatment.

We also tested the alternative hypothesis that an increased concentration of the REV1 inhibitors, beyond the 1 μM dose used above, would increase cytotoxicity with IR. To test this hypothesis, we treated IR-exposed HT1080 cells at 1 Gy to increasing concentrations of the drug JH-RE-06 at 5, 15, and 30 μM and measured relative increase in cytotoxicity. We observed that increasing the REV1 inhibitor’s concentration conferred a cytoprotective effect, where IR-exposed cells treated with 5 μM JH-RE-06 survived significantly better than the non-irradiated controls that received the drug alone (Figure 2B). The cells treated with JH-RE-06 alone, in fact, lost 80% of their viability, which was rescued in the IR-exposed group treated with JH-RE-06, suggesting that REV1 inhibition specifically during radiation treatment has a cytoprotective effect. These data suggested that REV1 inhibition propels intrinsic resistance during radiation treatment, directly contrasting its role in sensitizing cancer cells to chemotherapy. Alternatively, it is also possible that this observed effect is unique to the JH-RE-06 drug, where it slows general cell proliferation and perhaps exhibits an opposite effect to cytoprotection. Further work needs to be performed to establish this effect more clearly.

Because cancer cell lines over the course of cancer treatment tend to acquire resistance to therapy, we tested whether REV1 inhibition may sensitize acquired radioresistant cell lines to radiation treatment. To test this hypothesis, we treated isogenic pairs of cancer cells, including human breast cancer cells (ZR751 and its radioresistant counterpart ZR-751 RR) and canine mammary cancer cells (REM and its radioresistant counterpart REM RR) [19,20], with increasing doses of drug 5 (7587885), JH-RE-06, and JH-RE-06.NaOH at 1 and 10 μM. Our results demonstrated no significant differences in cytotoxicity in the radioresistant cell lines compared to their isogenic parental controls (Figure 2C and Figure S5). The REM RR radioresistant cells upon addition of 10 μM REV1 inhibitor JH-RE-06.NaOH displayed a slight growth advantage compared to the parental cell line. These results suggest that REV1 inhibition might also provide a cytoprotective effect in cancer cells that have acquired radiation resistance over the course of treatment.

To further verify if the lack of cytotoxicity from REV1 inhibition during radiation treatment was simply an IR-specific effect, we exposed HT1080 cells to etoposide (a cancer drug that functions like IR by inducing DNA strand-breakages and inducing cytotoxicity) and drugs 4 (7922759) and JH-RE-06. We did not observe any increase in synergy in cytotoxicity (Figure S6). Additionally, etoposide treatment did not sensitize the MEF REV1 KO cells, suggesting that REV1 inhibition perhaps engages a different biological response that enables cancer resistance during strand breakages from IR or etoposide treatment, which contrasts with the reduction in mutagenesis and chemoresistance upon REV1 inhibition during cisplatin treatment or treatment with other drugs that make chemical modifications to DNA.

### 2.2. REV1 Inhibition Triggers Autophagy, a Radioresistance Biomarker

**Figure 3:**
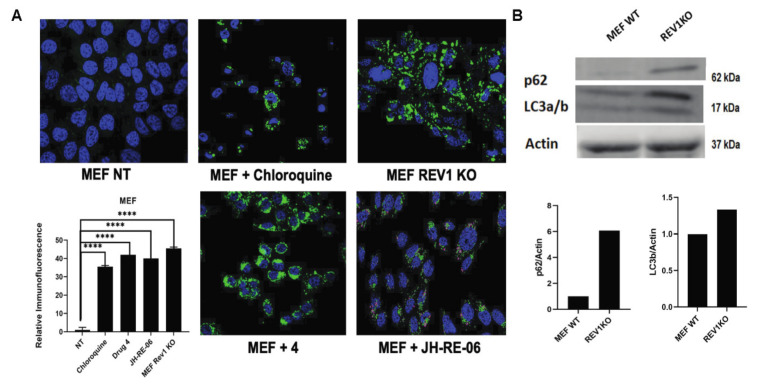
REV1 inhibition triggers autophagy. (**A**) Immunofluorescence images show autophagy flux (green) in MEF WT cells treated with chloroquine (positive control), drug 4 (7922759), and JH-RE-06, with REV1KO cells as validation controls. The graph shows relative quantification of the cells expressing the green fluorescence signal. *p*-Values are **** *p* < 0.0001. Error bars represent standard deviations. *p*-Values were calculated by *t*-test. *N* ≥ 50. Images are at 40×. (**B**) Representative image of a Western blot showing expression of p62 and LC3a in MEF REV1 KO cells compared to the WT MEF. Graph shows relative quantification.

**Figure 4:**
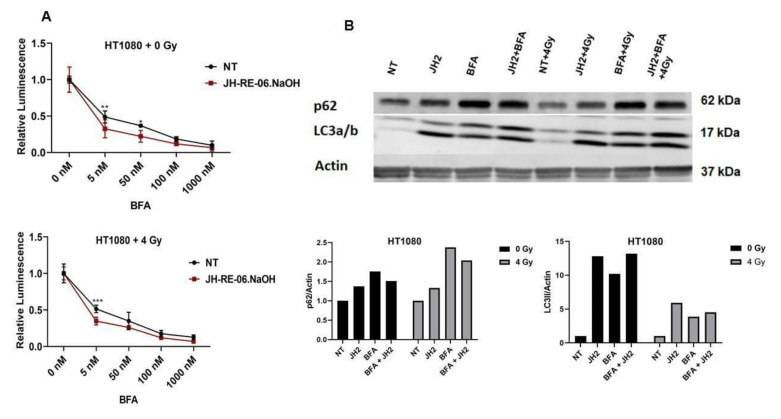
Autophagy inhibition has a narrow range for sensitizing cancer cells treated with ionizing radiation and REV1 inhibitors. (**A**) Graphs show relative luminescence in HT1080 cells treated with 0 or 4 Gy ionizing radiation in the presence of increasing doses of autophagy inhibitor BFA and 1 uM of JH-RE-06.NaOH. (**B**) Representative Western blot images show expression patterns of p62 and LC3a/b in HT1080 cells treated with JH-RE-06.NaOH at 1 mM, BFA at 50 mM, and ionizing radiation at 0 and 4 Gy, respectively. Graphs show relative quantification of p62 and LC3a/b expression in HT1080 cells from the Western blots above. *p*-Values are * *p* < 0.05, ** *p* < 0.01, and *** *p* < 0.001. Error bars represent standard deviations. *p* values were calculated by 2-way ANOVA. *N* = 6 for all values.

During our experiments, we observed an unusual phenotype of the MEF REV1 KO cells (Figure S7), where the cells appeared flattened under an inverted microscope as previously reported [8]. However, we also observed a curious cellular morphology of enlarged cytoplasmic vesicles that prompted us to test whether the enlarged structures were lysosomes [21]. Enlarged lysosomes are typically discernible with differential interference contrast (DIC) brightfield microscopy. Next, we used the Cyto-ID green detection reagent (Enzo Life Sciences), a cationic dye with strong affinity for pre-autophagosomes, autophagosomes, and autophagolysosomes that are typically upregulated during autophagy. Over 40% of MEF REV1 KO cells exhibited marked staining suggestive of autophagy induction in the absence of REV1 (Figure 3A). Because MEF REV1 KO cells grow slower than the MEF WT cells, we wanted to test whether growth dynamics influenced autophagy induction [8]. To test this hypothesis, we used REV1 inhibitors, drugs 4 (7922759) and JH-RE-06, at a 1 μM concentration to confirm the specificity of REV1’s role in autophagy induction. We observed approximately 50% cell staining with the Cyto-ID green detection reagent (Figure 3A and Figure S8). The induction of autophagy from the two REV1 inhibitors in these immunofluorescence assays was equivalent to or stronger than (more than 70% increase in lysosomal staining) that of the positive control, chloroquine, and MEF REV1 KO cells, suggesting that REV1 inhibition triggers autophagy (Figure 3A). In addition to staining phagosomal structures that mark induction of autophagy, increased LC3A/B protein ratios also successfully signal autophagy induction. LC3B is one of the three isoforms of the protein LC3. During autophagy, LC3A undergoes lipidation and converts to LC3B, which then associates with autophagosomes as part of the autophagy process [22,23]. Western blot analysis of the MEF REV1 KO cells showed an almost a 1.5-fold increased LC3B protein expression compared to their normal MEF cells, but we also observed a curious phenotype of an increased expression of LC3A exclusively in the MEF REV1 KO cells (Figure 3B). The MEF REV1 KO cells also exhibited an up to six-fold increase in p62 or SQSTM1 expression, which is another readout of the autophagy flux. The relative gene expression quantification from one representative image from two independent experiments is shown under the Western blot images. Similarly, exposure of the HT1080 cells to REV1 inhibitor, JH-RE-06.NaOH, increased the LC3B levels by 10-fold and the p62 levels by about 1.5-fold, as compared to non-treated controls (Figure 4B). Both these results indicate that REV1 inhibition triggers autophagy. It is interesting to note that the REV1 KO cell line upregulates LCA3, while the use of REV1 inhibitors increases the expression of LC3B. Future studies must address these unique differences in autophagy induction by genetic perturbation of the key gene versus the use of small molecule inhibitors to target gene function.

Autophagy is known to cause resistance to IR therapy in radioresistant cancer cells [13]. The context within which REV1 inhibition plays into the induction or the maintenance of autophagy signals in cells is unknown. Similarly, whether REV1 inhibition-mediated radioresistance could be suppressed by inhibiting autophagy is not known. To test the latter hypothesis, we used the autophagy inhibitor bafilomycin A1 (BFA) that targets the autophagosome–lysosome fusion to inhibit autophagy [24]. We treated HT1080 cells with increasing doses of BFA to establish the dose range at which it sensitizes JH-RE-06.NaOH-treated cells to ionizing radiation. Dose–response curves suggest that the optimal dose to inhibit REV1-inhibition induced autophagy is a concentration of 5 nM of BFA in both IR-exposed cells and controls (Figure 4A). Higher doses of BFA triggered its own autophagy response, as can be seen by the increase in toxicity in the dose–response curves. To verify that higher concentrations of BFA triggered autophagy and consequently toxicity in our dose–response curves, we ran Western blots of IR and REV1 inhibitor JH-RE06.NaOH-treated cells which were exposed to 50 nM of BFA and observed an induction of both LC3B and p62 (Figure 4B). These assays suggest that there is only a narrow range within which an autophagy inhibitor can potentially synergize cells to radiosenitization post-REV1 inhibition. Further studies are needed to systematically analyze the association of REV1 with autophagy and the utility of other autophagy inhibitors. However, because a functional reduction of REV1 was key in triggering the autophagy and the inhibition of REV1 failed to radiosensitize cells, REV1 expression dynamics may link cancer cell response to radiation treatment through the potential induction of autophagy.

## 3. Discussion

In the last few years, the translesion synthesis pathway, especially the REV1 polymerase, has gained considerable traction in understanding how cancers acquire intrinsic and acquired resistance to therapy. The function of REV1, which was classically known to facilitate the formation of new mutations by functioning as a deoxycitidyl transferase, is also now known to allow protein–protein interactions with other TLS polymerases by folding into a scaffolding molecule [1]. These landmark observations provide a semblance as to why the cancer mutational spectrum continues to evolve and an understanding as to why there remains a continuity to clinical challenges in treating patients. Furthermore, the discovery of REV1 inhibitors provided a reliable platform for potential clinical adjuvant therapy [2,3,4,5]. However, indications that REV1 inhibition can also switch the underlying biology of cancer cells, whereby it can suppress apoptosis in cisplatin-treated cells and trigger senescence [8], suggested that REV1 may have a larger new role in cancer pathogenesis.

This study shows two unexpected observations that further our understanding of REV1 functional dynamics during cancer resistance and the consequences of targeting REV1 during different DNA-damaging cancer treatments. First, we found that REV1 inhibition during radiation therapy may not sensitize cancer cells to increased cell death, as indicated by our in vitro data. In colony survival assays we observed that REV1 inhibition with five different REV1 inhibitors did not sensitize cells to IR. Neither increased exposure times to REV1 inhibitors nor increased concentrations of REV1 inhibitors during IR resulted in a synergy in cytotoxicity. Additionally, acquired radioresistant cancer cells REM and ZR751, developed by exposing the parental cells to repeat rounds of IR, survived similarly to non-treated controls when exposed to REV1 inhibitors. In summary, our data suggest that REV1 inhibition does not sensitize cancer cells with intrinsic and acquired resistance to radiation treatment. This contrasts with an earlier study in which siRNA depletion of REV1 in HeLa cells resulted in enhanced radiosensitivity [25]. However, it has also been shown that genetic heterogeneity of HeLa cells can lead to changes in protein expression and could impact the interpretation of results obtained from this cell line [26,27]. The data presented from our research were obtained using three different cell lines and different REV1 inhibitors. Despite this, we did not see any enhanced radiosensitization. It is possible that these differences arise due to differences in cell lines or the method of inhibiting REV1 function. It is also noted that the small molecule inhibitors of REV1 that were used were effective in chemosensitization as reported earlier, but not in radiosensitization, as observed in this study.

Additionally, REV1 inhibition did not sensitize etoposide-treated cancer cells, suggesting that treatment modules that rely on DNA strand breaks to trigger cell death in cancer cells may not benefit from inhibiting REV1, unlike the significant potential of REV1 inhibition to sensitize cancer cells to chemotherapy treatment. That is, depending upon the type of DNA-damaging cancer treatment chosen, levels of functional REV1 may help enhance the efficacy of the said drug, as would be the case for chemotherapy treatment that causes chemical modifications to DNA, where REV1 contributes to mutagenesis and consequent therapy resistance. In contrast, a similar reduction in functional REV1 during radiation treatment or drugs that cause strand breaks in cancer cells will propel therapy resistance because REV1 inhibition evokes a newer function of induction of autophagy. This result further suggests that REV1 may be an essential biomarker for cancer treatment success, whereby its reduced levels during chemotherapy would be an indicator of good patient response to treatment. On the other hand, increased levels would be desired for patients undergoing radiotherapy. Further, the REV1 expression profile across 31 tumor samples and paired normal tissues shows that almost 50% of these tumor tissues exhibited reduced expression of REV1 (http://gepia.cancer-pku.cn/detail.php?gene=REV1, accessed on 9 September 2021).

We also show the first direct evidence of REV1 inhibition-dependent activation of the autophagy flux, an uncharacterized biological consequence previously unknown to be associated with the REV1 translesion synthesis polymerase. We observed marked cytoplasmic vesicle formation in MEF REV1 KO cells that were stained as autophagosomes. Further, the induction of autophagosomes after REV1 inhibition in independent cell lines suggested that REV1 has an unanticipated role in regulating the autophagy stress response. Moreover, because we observed an upregulation of the LC3B isoform of LC3 in both the MEF REV1 KO and JH-RE-06-exposed HT1080 cells, REV1 may be an active modulator of autophagy.

Typically, autophagy is a potent mechanism triggered to combat the consequences of starvation stress, the accumulation of damaged cellular components, and in certain instances to promote cancer resistance. Besides, a dominant autophagic flux in certain circumstances can induce senescence in cancer cells [28]. Several questions remain unaddressed: How might REV1, a translesion synthesis polymerase, fit within the complex interplay of autophagy induction? In addition, how does REV1 serve to engage cellular responses such as autophagy versus regulating DNA repair versus senescence induction during IR-induced DNA strand damage? REV1 inhibition was previously shown to trigger senescence in chemotherapy-treated cells [8]. It is equally intriguing to evaluate whether REV1 might serve as a clinical biomarker for cancer cell response to other cancer treatments. For example, it could be studied whether patients with higher REV1 expression levels respond better to radiation treatment as compared to those with lower REV1 expression levels, where autophagy induction may result in a poor response to radiation treatment.

This study collectively shows that REV1 inhibition confers a cytoprotective effect on cancer therapies aimed at inducing DNA strand breakage. We also observed that REV1 inhibition induces autophagy, a known biomarker of radioresistance. Further work needs to be performed to determine a connection between REV1 inhibition, the induction of autophagy, and radioresistance.

## 4. Methods

### 4.1. Mammalian Cell Culturing

HT1080 cells (male, fibrosarcoma epithelial cells purchased from ATCC, Manassas, VA, United States) were grown at 37 °C with 5% CO_2_ in EMEM (ATCC) and RPMI (ATCC), 10% (*v*/*v*) FBS (Gibco, Amarillo, TX, USA), and 1% penicillin–streptomycin antibiotic (Gibco). HCT116 cells (male, colorectal epithelial cancer cells purchased from ATCC) were grown in McCoy’s 5A (ATCC) 10% (*v*/*v*) FBS (Gibco), and 1% penicillin–streptomycin antibiotic (Gibco). Mouse embryonic fibroblasts (MEF) along with their REV1 knockout counterparts were grown at 37 °C with 5% CO_2_ in DMEM (ATCC), 10% (*v*/*v*) FBS (Gibco), and 1% penicillin–streptomycin antibiotic (Gibco). REM (canine mammary cancer cells), ZR751 (human breast cancer cell line), and their radioresistant counterparts REM RR and ZR751 RR 10 were graciously donated by Mark Gray, University of Edinburgh. These cells were grown at 37 °C with 5% CO_2_ in DMEM (ATCC), 10% (*v*/*v*) FBS (Gibco), and 1% penicillin–streptomycin antibiotic (Gibco). 0.25% trypsin was used for trypsinizing and splitting.

### 4.2. Drug Inhibitors

REV1 inhibitors targeting the 2 interfaces of the C-terminus domain (CTD)—drugs 4 (7922759), 5 (4053831), 6 (7127492) targeting the RIR (REV1 interacting region) interface, and JH-1 (JH-RE-06) and JH-2 (JH-RE06.NaOH) targeting the REV7 interface—were used in this study [3,4,5]. Bafilomycin A1 (BFA), a macrolide antibiotic that inhibits late-phase autophagy, was used an autophagy inhibitor (Sigma Cat # B1739, Tokyo, Japan).

### 4.3. Cytotoxicity Assay

A total of 10,000 cells were plated into each well of a white-bottom 96-well plate (Corning, New York, NY, USA). The cells were treated with varying concentrations (1, 5, 15, or 30 μM) of REV1 inhibitor drugs (JH-RE-06, JH-RE-06.NaOH, drug 4, drug 5, drug 6) with either 0, 1, or 4 Gy of ionizing radiation. After 24, 48, or 72 h of incubation, the proportion of viable cells was evaluated by adding 100 µL of the CellTiter-Glo Luminescence stain (Promega, Madison, WI, USA) was added into each well. The CellTiter Glo stain was prepared according to the manufacturer’s recommendation. The endpoint luminescence was measured on the Synergy H1 Microplate Reader plate reader. Relative cell viability was determined by dividing treated sample luminescence measurements by their respective control samples without drugs and/or ionizing radiation.

### 4.4. Colony Survival Assay

Approximately 600 to 800 cells were plated in triplicate into each well of 6-well plates for 24 h. The cells were treated with 1 µM of various REV1 inhibitor drugs (JH-RE-06, JH-RE-06.NaOH, drug 4, drug 5, drug 6) for 24 h and exposed to varying levels of ionizing radiation (0, 1, or 4 Gy). The plates were incubated at 37 °C for 24 h, after which media were replaced with fresh media and plates were incubated at 37 °C for 6–7 days. The media was aspirated, and the cells were fixed with 70% ethanol before staining with 1 mL of 0.1% crystal violet dye. Stained colonies containing at least 40 cells were counted and relative cell survival was quantified by dividing the average number of colonies from each condition by the average of their respective negative controls (no IR and/or no drugs).

### 4.5. Immunofluorescence Detection of Autophagy

MEF and HT1080 cells were plated in 35 mm dishes for 24 h, after which they were treated with a 1 μM concentration of drug 4 and JH-RE-06 for another 24 h. Cells were then fixed with 4% paraformaldehyde, and immunofluorescent detection of autophagy was assessed using the CYTO I.D. Autophagy Detection Kit 2.0 (Enzo Life Sciences; Catalog number: ENZ-KIT175-0050, Farmingdale, New York, NY, USA). The kit is optimized to detect autophagic vacuoles as well as autophagic flux in lysosomal-inhibited live cells. A fluorescent green dye labels autophagic vacuoles as they accumulate. Bright green fluorescence is indicative of autolysosomes. The stained samples were imaged at the UVM Cancer Center’s Microscope Imaging Center (MIC).

### 4.6. Western Blot Analysis

Cells were lysed in RIPA lysis buffer (Pierce, Waltham, MA, USA) with fresh protease inhibitors (Pierce), and the lysate was quantified using the Micro BCA Protein Assay Kit (Pierce). Samples were boiled for 5 min in 4x LDS Sample Buffer (Invitrogen, Waltham, MA, USA) and separated by SDS-PAGE. Separated proteins were transferred to polyvinylidene difluoride membrane using the Mini Trans-Blot Electrophoretic Transfer Cell (BioRad, Hercules, CA, USA) for 90 min at 100 V. The following antibodies were used in Blocking Buffer (Thermo Scientific, Waltham, MA, USA): LC3A/LC3B (Invitrogen PA1-16931) at 1:500, SQSTM1 (Invitrogen PA1-27247) at 1:500, γH2AX (Novus NB100-384) at 1:1000, and Actin (Invitrogen MA1-744) at 1:1000. The membrane was washed with dPBST (Corning, +0.1% Tween) 3 times for 15 min each and incubated with secondary antibody (LC3A/B/γH2AX/p62: IRDye 800CW, Goat Anti-Rabbit; Actin: IRDye 680RD, Goat Anti-Mouse) at a 1:20,000 dilution in blocking buffer with 0.01% SDS and 0.1% Tween-20. Blots were visualized using a LiCOR imager and analyzed with Image Studio software.

Image studio was used to quantify relative expression of proteins in the Western blots by dividing the A.U. for key genes with the corresponding actin control and were subsequently normalized to their respective experimental controls. Original blots can be found at Figures S9–S14.

### 4.7. Statistical Analysis

Statistical analysis was carried out by 2-way analysis of variance (ANOVA). Each result was the sum of at least 2 biological replicates, with *n* = 6 in most cases, unless otherwise noted. Standard deviations (S.D.) indicate the variance and are indicated as the mean ± S.D., unless otherwise noted. Significance is noted as * *p* < 0.05, ** *p* < 0.01, *** *p* < 0.001, or **** *p* < 0.0001.

## 5. Conclusions

In this study, we hypothesized that REV1 inhibition would sensitize cells to radiation therapy. We observed that REV1 inhibition does not sensitize cancer cells to ionizing radiations, where a combination treatment of different REV1 inhibitors and radiation treatment failed to sensitize HT1080, HCT116, and MEF cells. In addition, increasing the dose or the incubation time with REV1 inhibitors did not sensitize cells to radiation treatment. Conversely, autophagic cell organelles were demonstrated in REV1-inhibited cells by immunofluorescence, and autophagy induction was confirmed in western blot analysis by the increased expression of autophagy proteins LC3 and SQSTM1 in drug inhibited cells and the REV1KO cells. This study also reports a narrow window within which an autophagy inhibitor, Bafilomycin A1 (BFA), might sensitize REV1-inhibited cells to killing with radiation. In conclusion, this study suggests that inhibition of the TLS polymerase, REV1, may not be an excellent synergistic approach to treating cancer cells with radiation due to a possible induction of autophagy.

## Data Availability

The data presented in this study are available on request from the corresponding author.

## Supplementary Materials

The following are available online at https://www.mdpi.com/article/10.3390/cancers13215290/s1, Figure S1: Structures of drugs used in this study, Figure S2: Relative colony survival post-treatment with drugs 5 and 6. Figure S3: Relative viability in cytotoxicity assays from different drug treatments. Figure S4. Relative viability in cytotoxicity assays from increasing exposure time to REV1 inhibitor JH-RE-06 during IR treatment. Figure S5: Relative viability in cytotoxicity assays of radioresistant cell lines from increasing exposure to REV1 inhibitors. Figure S6: Relative viability in cytotoxicity assays from REV1 inhibition and etoposide treatment. Figure S7: MEF REV1 KO cells exhibit large translucid intracytoplasmic vacuoles and autophagy. Figure S8: REV1 inhibition triggers autophagy in HT1080 cells. Figure S10–S14: Original blots.
