# Supplementary material for: REV1 Inhibition Enhances Radioresistance and Autophagy
Source: Cancers (Basel). 2021 Oct 21;13(21):5290. doi: 10.3390/cancers13215290 (PMC8582445; doi:10.3390/cancers13215290)
Supplement: Supplementary file 1 [file cancers-13-05290-s001.zip › cancers-1394725-supplementary.pdf]

Supplementary Materials

REV1 Inhibition Enhances Radioresistance and Autophagy

Kanayo E. Ikeh, Erica N. Lamkin, Andrew Crompton, Jamie Deutsch, Kira J. Fisher, Mark Gray, David J. Argyle, Won Y. Lim, Dmitry M. Korzhnev, M. Kyle Hadden, Jiyong Hong, Pei Zhou and Nimrat Chatterjee

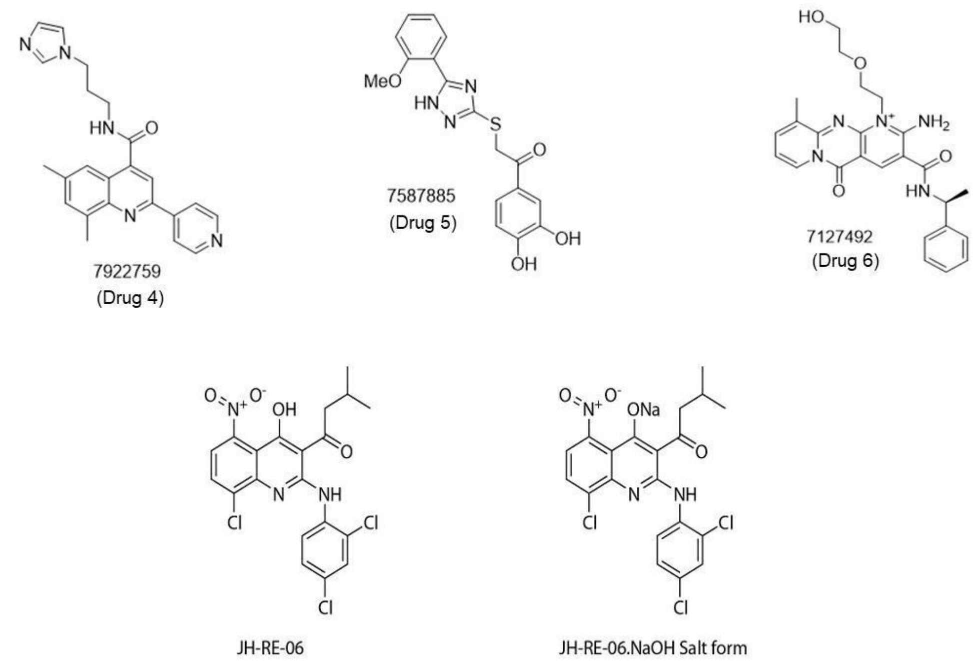

Figure S1. Structures of drugs used in this study: 4 (7922759), 5 (7587885), 6 (7127492), JH-1 (JH-RE-06) and JH-2 (JH-RE06.NaOH) via the CYTO-ID green detection reagent kit (Enzo Life Sciences).

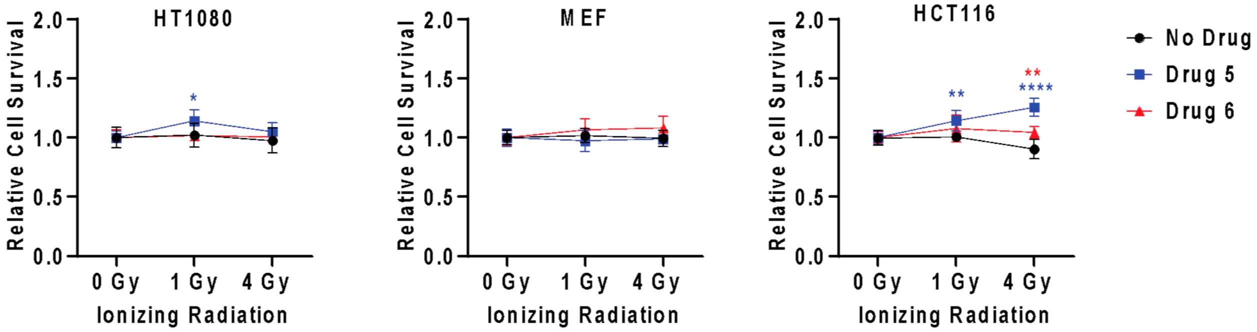

Figure S2. Relative colony survival post-treatment with drugs 5 and 6. Charts show colony survival assays post-REV1 inhibition using drugs 5 (7587885) and 6 (7127492) with increasing exposure to IR treatment (0, 1, and 4 Gy), with some cytoprotection in HT1080 and HCT116. P values are *P < 0.05, ** P < 0.01, and **** P < 0.0001. Error bars represent standard deviations. P values were calculated by two-way ANOVA. *N* = 6 for all values.

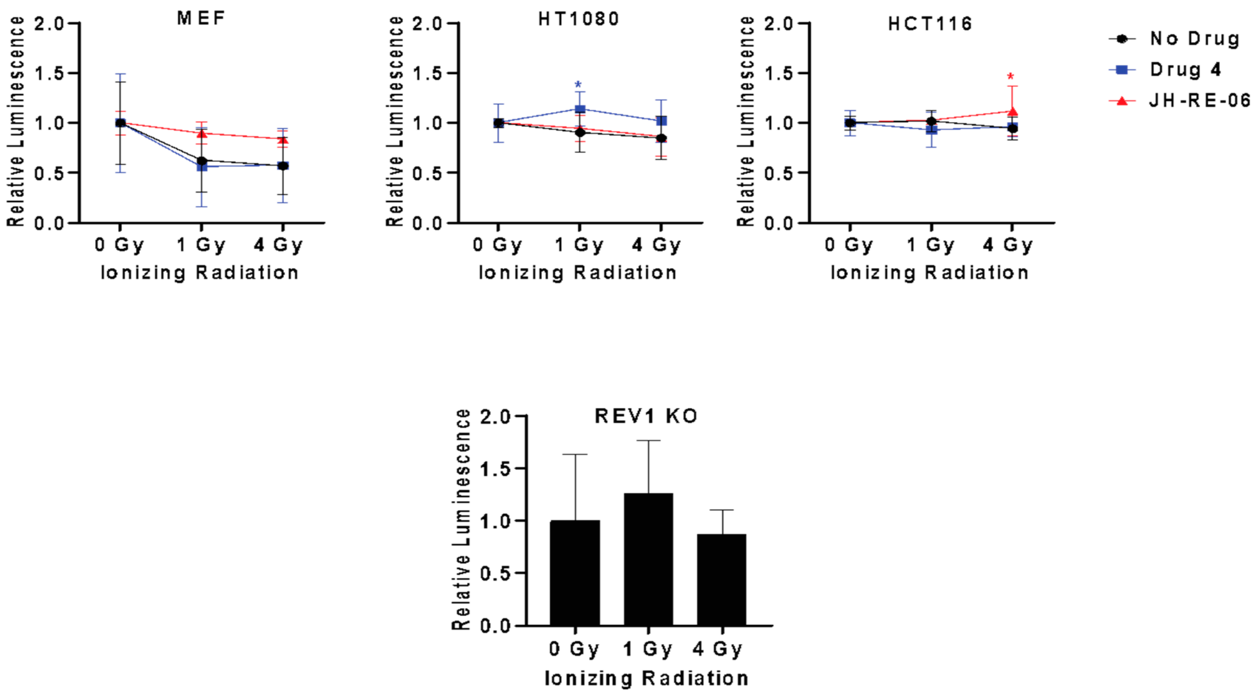

Figure S3. Relative viability in cytotoxicity assays from different drug treatments. REV1 inhibition by REV1 inhibitor drugs 4 (7922759) and JH-1 (JH-RE-06), does not sensitize cancer cells to IR exposure. Shown here are graphs measuring relative luminescence in MEF, HT1080, HCT116 and MEF REV1KO cell lines upon exposure with increasing doses of IR (0, 1 and 4 Gy). P values are *P<0.05. Error bars represent standard deviations. P values were calculated by two-way ANOVA. *N* = 6 for all values.

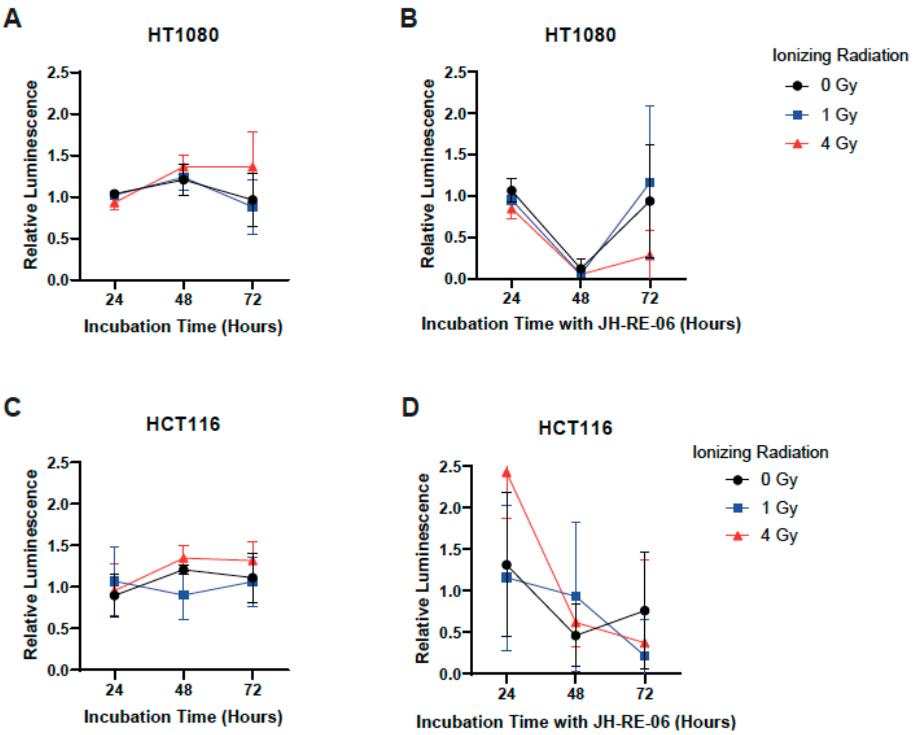

Figure S4. Relative viability in cytotoxicity assays from increasing exposure time to REV1 inhibitor JH-RE-06 during IR treatment. Graphs demonstrate relative luminescence intensities measured at 24, 48, and 72-hours post-treatment with 0, 1 and 4 Gy of IR and JH-RE-06 drug in HT1080 and HCT116 cell lines. (**A**,**C**) are no treatment controls. (**B**,**D**) contained the JH-RE-06 REV1 inhibitor. Error bars represent standard deviations. P values were calculated by two-way ANOVA. *N* = 4-6 between different experimental conditions.

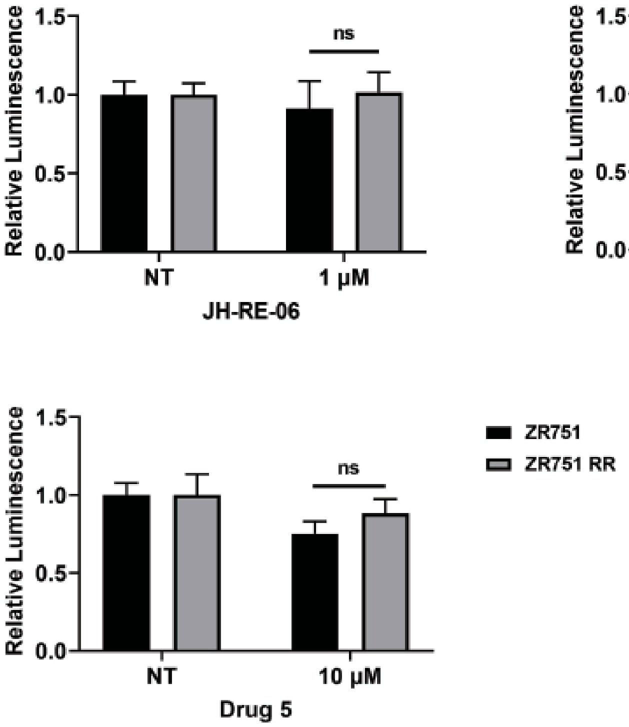

Figure S5. Relative viability in cytotoxicity assays of radioresistant cell lines from increasing exposure to REV1 inhibitors. Shown here are relative luminescence results for REM and REM RR cell lines treated with 1 µM JH-RE-06 and 10 µM of JH-RE-06.NaOH; and ZR751 and ZR751 RR treated with 1 µM JH-RE-06. Error bars represent standard deviations. P values were calculated by two-way ANOVA. *N* = 6 for all values.

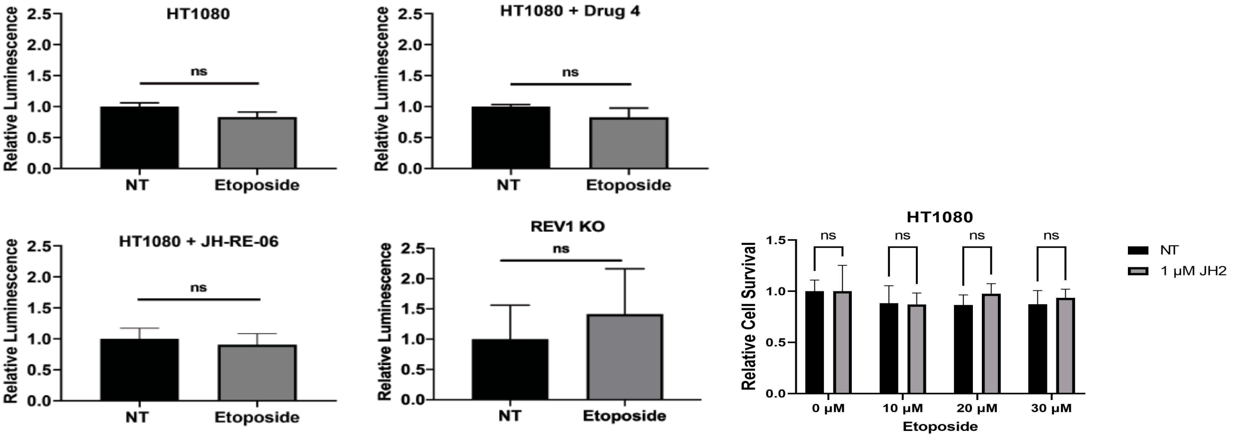

Figure S6. Relative viability in cytotoxicity assays from REV1 inhibition and etoposide treatment. Graphs demonstrate the relative luminescence of HT1080 and REV1 KO cells upon treatment with REV1 inhibitor drugs and etoposide. REV1 inhibition using drug 4 (7922759) and JH-RE-06 does not sensitize cells to treatment with etoposide at 10 µM. Error bars represent standard deviations. P values were calculated by two-way ANOVA. *N* = 6 for all values.

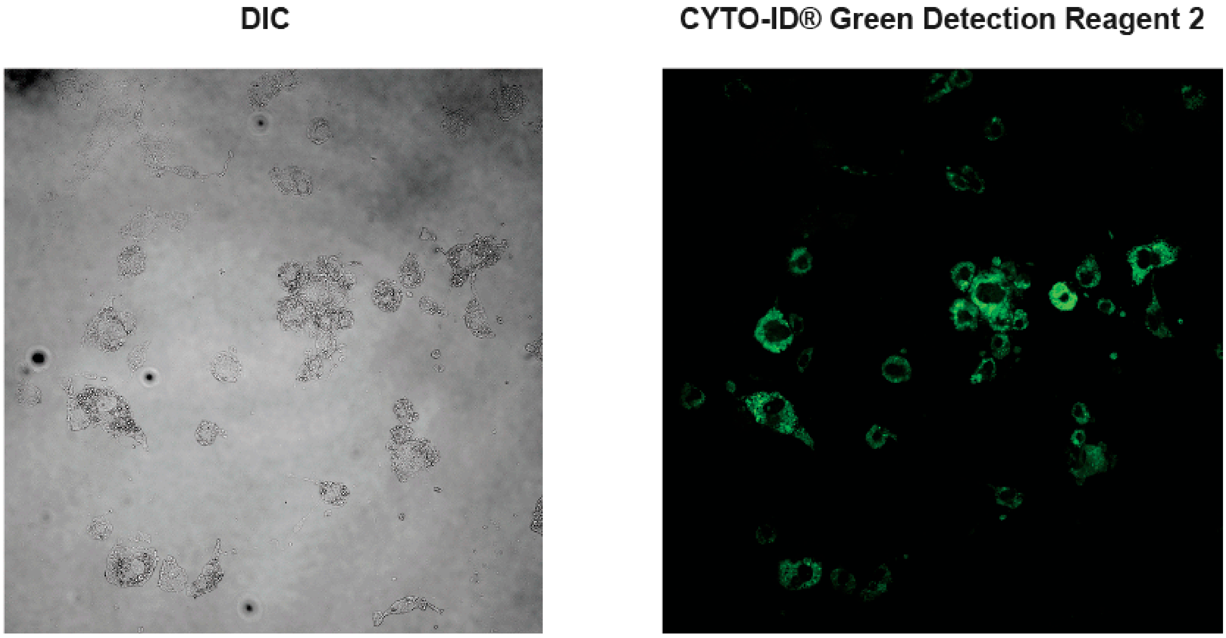

Figure S7. MEF REV1 KO cells exhibit large translucid intracytoplasmic vacuoles and autophagy. The inverted light microscopy images of MEF REV1 KO cells on the left and green fluorescence signal from staining of autophagosomes via the CYTO-ID green detection reagent kit (Enzo Life sciences). Images are at 40×.

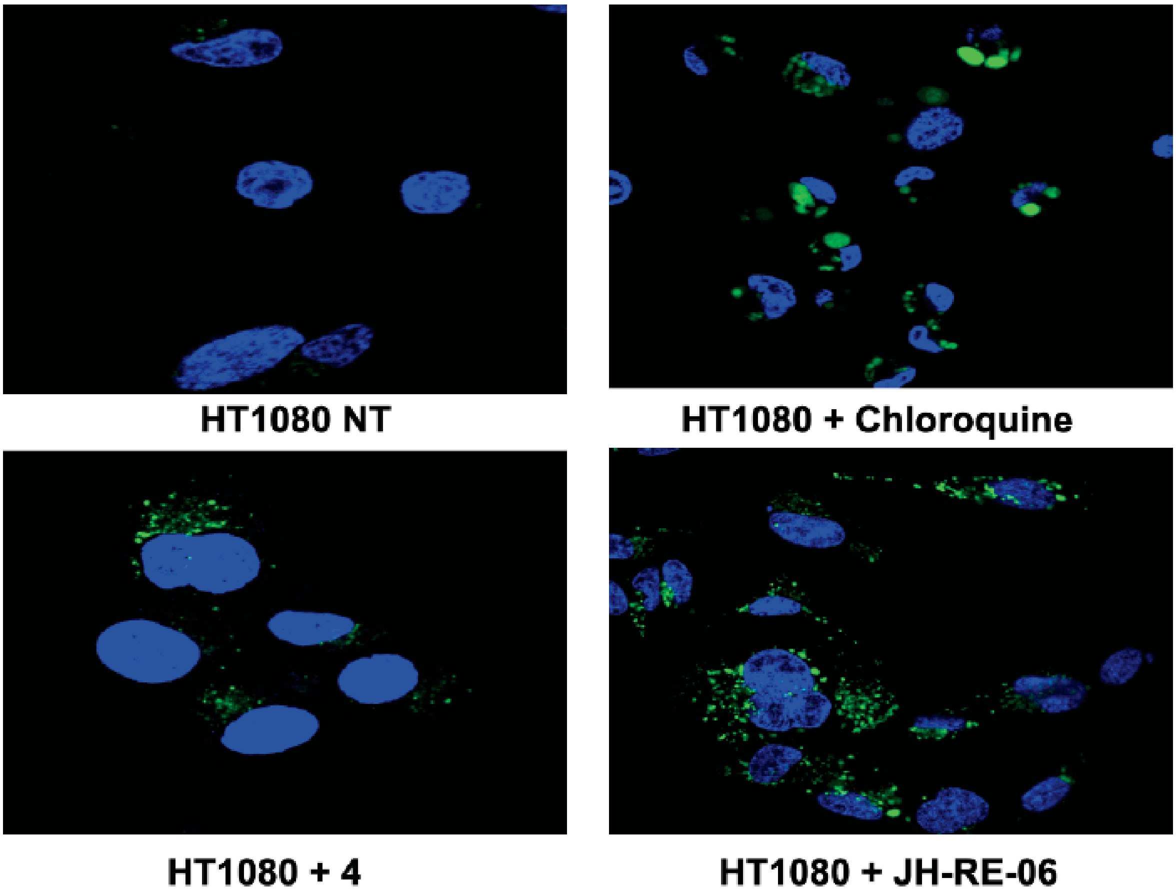

Figure S8. REV1 inhibition triggers autophagy in HT1080 cells. Shown here are immunofluorescence images of HT1080 cells treated with chloroquine (positive control), drug 4 (7922759), and JH-RE-06. Inhibition of REV1 function by drug 4 and JH-RE-06 triggers autophagy (green signal). The green fluorescence signal is from staining of autophagosomes. Images are at 40×.

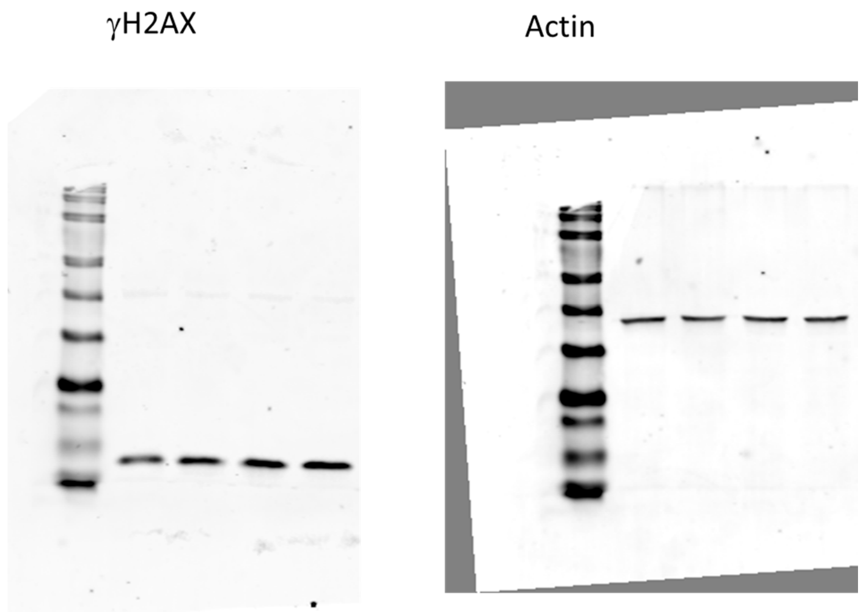

Figure S9. Full images- Fig. 1D.

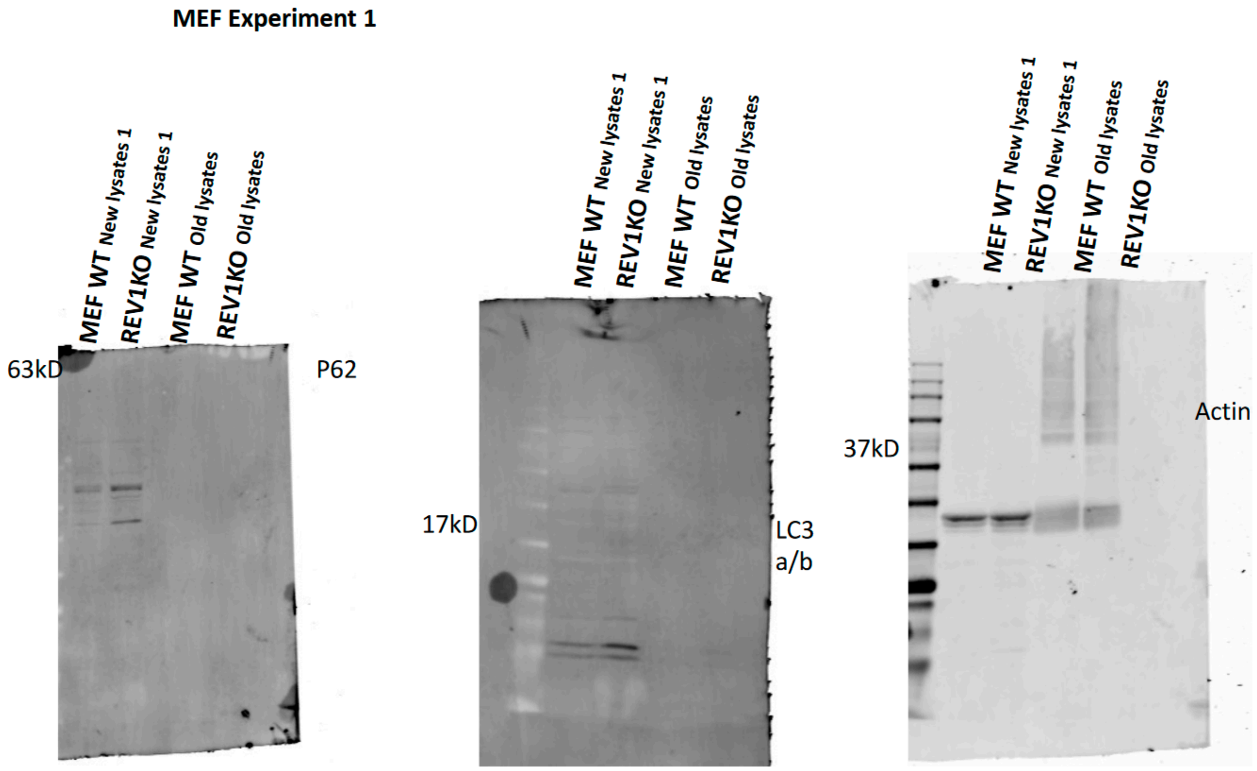

Figure S10. Original blots for Figure 3B.

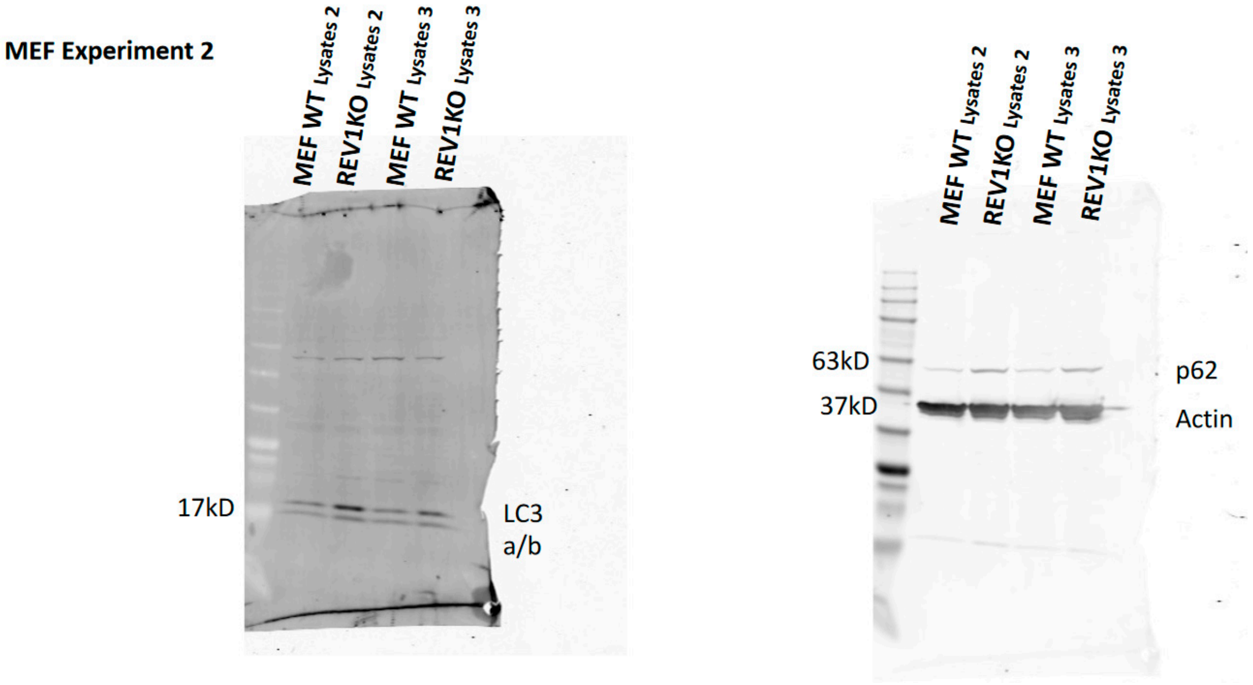

Figure S11. Original blots for Figure 3B.

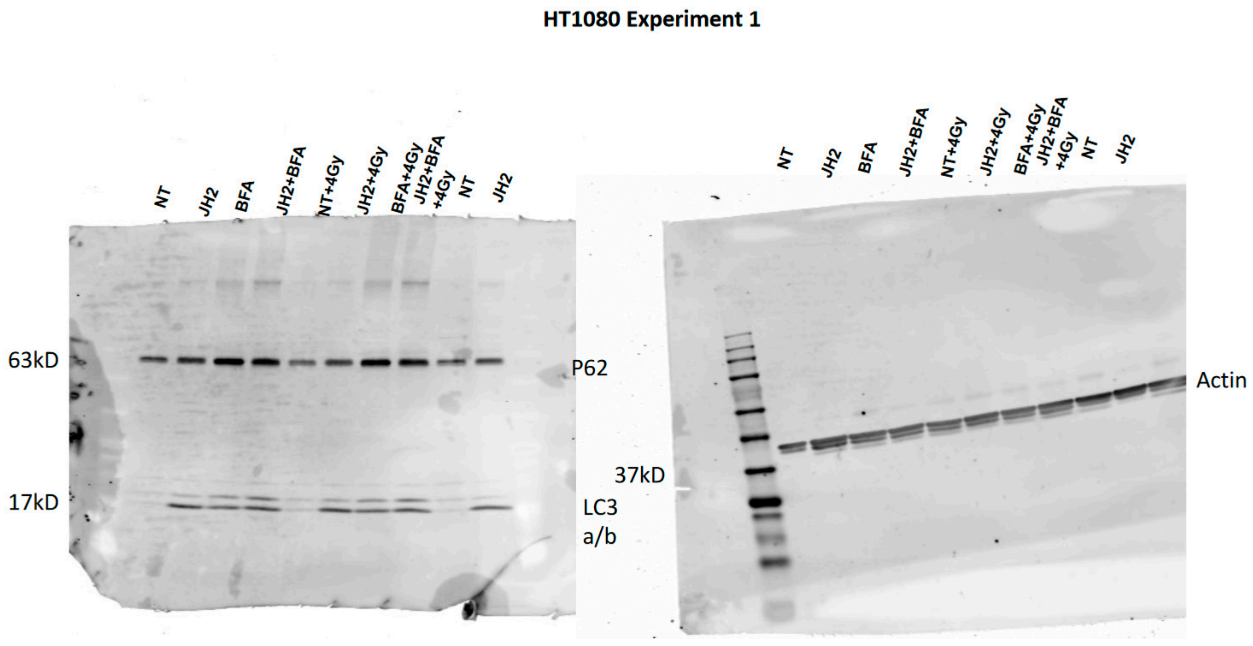

Figure S12. Original blots for Figure 4B.

HT1080 Experiment 2

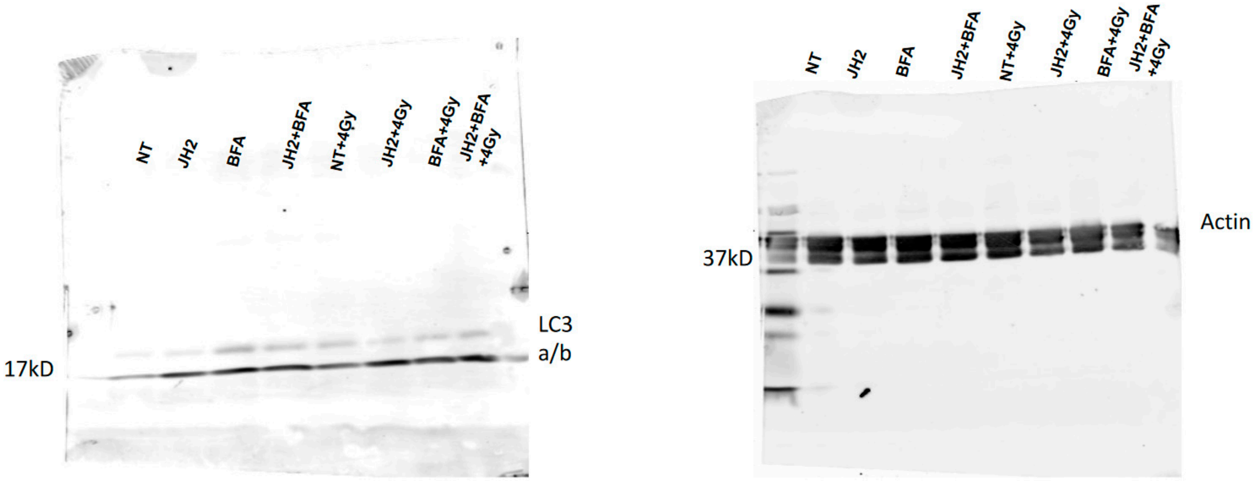

Figure S13. Original blots for Figure 4B.

HT1080 Experiment 2

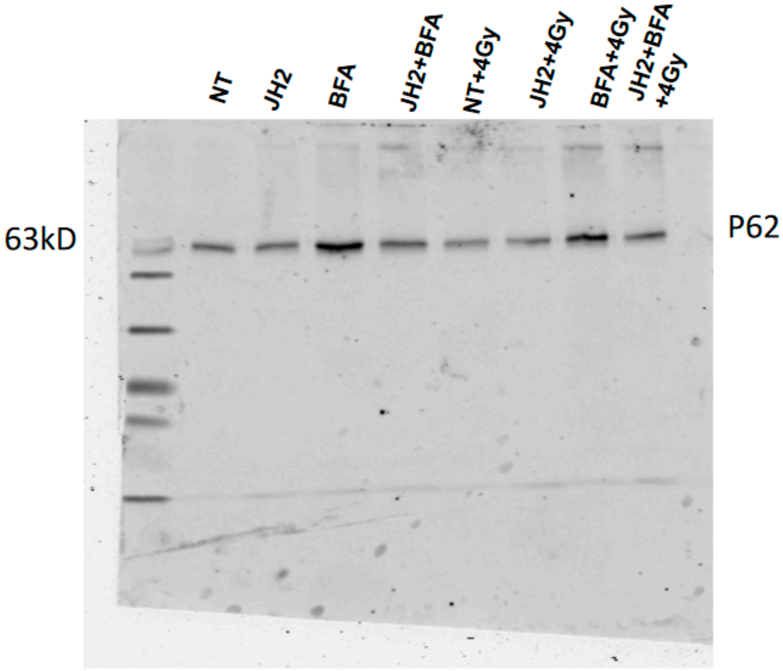

Figure S14. Original blots for Figure 4B.
